# Experimental study of tuberculosis: From animal models to complex cell systems and organoids

**DOI:** 10.1371/journal.ppat.1006421

**Published:** 2017-08-17

**Authors:** Kaori L. Fonseca, Pedro N. S. Rodrigues, I. Anna S. Olsson, Margarida Saraiva

**Affiliations:** 1 i3S - Instituto de Investigação e Inovação em Saúde, Universidade do Porto, Porto, Portugal; 2 IBMC - Instituto de Biologia Molecular e Celular, Universidade do Porto, Porto, Portugal; 3 ICBAS - Instituto de Ciências Biomédicas Abel Salazar, Universidade do Porto, Porto, Portugal; Stony Brook University, UNITED STATES

## Abstract

Tuberculosis (TB) is a devastating disease to mankind that has killed more people than any other infectious disease. Despite many efforts and successes from the scientific and health communities, the prospect of TB elimination remains distant. On the one hand, sustainable public health programs with affordable and broad implementation of anti-TB measures are needed. On the other hand, achieving TB elimination requires critical advances in three areas: vaccination, diagnosis, and treatment. It is also well accepted that succeeding in advancing these areas requires a deeper knowledge of host—pathogen interactions during infection, and for that, better experimental models are needed. Here, we review the potential and limitations of different experimental approaches used in TB research, focusing on animal and human-based cell culture models. We highlight the most recent advances in developing in vitro 3D models and introduce the potential of lung organoids as a new tool to study *Mycobacterium tuberculosis* infection.

## Author summary

Tuberculosis (TB) is the number 1 killer in the world due to a bacterial infection. The study of this disease through clinical and epidemiological data and through the use of different experimental models has provided important knowledge on the role of the immune response generated during infection. This is critical for the development of novel vaccines and therapeutic strategies. However, in spite of the advances made, it is well accepted that better models are needed to study TB. This review discusses the different models used to study TB, highlighting the advantages and disadvantages of the available animal and cellular models and introducing recently developed state-of-the-art approaches based on human-based cell culture systems. These new advances are integrated in a road map for future study of TB, converging for the potential of lung organoids in TB research.

## General introduction

**Fig 1:**
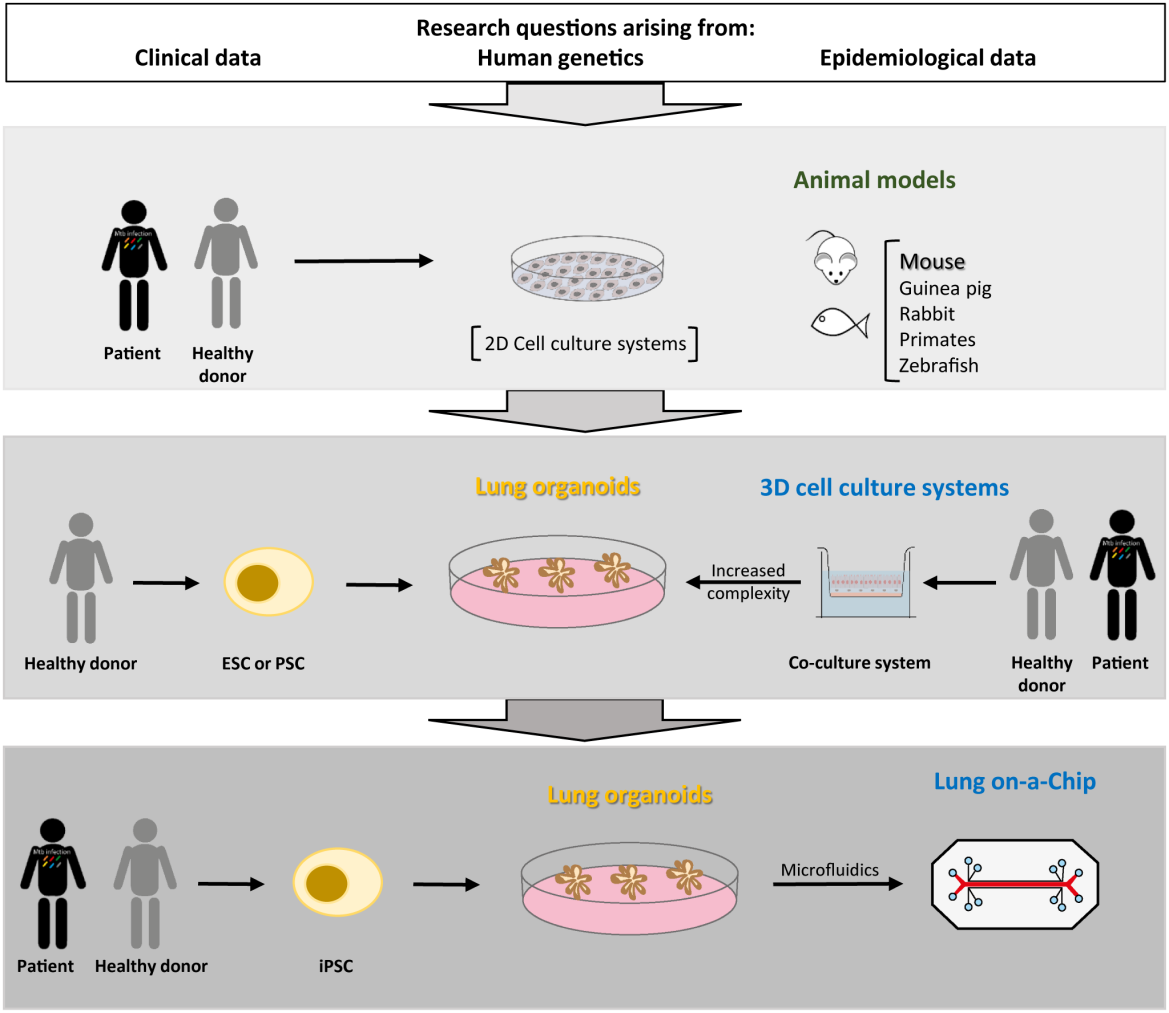
An integrative view on the experimental models for tuberculosis (TB) research. Questions arising from clinical, genetic, and epidemiological data on TB are addressed using a variety of experimental approaches. The traditional approach has used a combination of 2D culture systems and animal models. The recent development of 3D cell culture systems composed by multiple cell types provides in vitro models with a level of complexity previously only available in vivo. The generation of lung-on-chip cultures and the possibility of generating lung organoids from healthy or patient donors may in future offer experimental systems closer to the human pathophysiology. **Abbreviations:** ESC, embryonic stem cells; iPSC, induced pluripotent stem cells; PSC, pluripotent stem cells.

Tuberculosis (TB) kills over 1.8 million people every year and thus remains the leading cause of death by an infectious agent [1]. Additionally, TB afflicts over 10.4 million new individuals per year and is estimated to exist in a latent form in nearly 2 billion people worldwide [1]. In addition to the human toll, TB imposes a significant economic burden, corresponding to 0.52% of the global gross national product, with a cost of over 500 million euros per year in the European Union alone [2]. Tackling TB is therefore a matter of urgency, as reflected in the current WHO End TB Strategy, which targets a 90% reduction in the incidence of TB to less than 100 cases per million people by 2035 [3]. Achieving this target requires a much quicker decline in TB incidence, from the current annual reduction of 2% to a 20% decrease per year [4, 5]. For this, 3 areas in TB research are generally accepted as critical: development of novel vaccines, improved diagnostic tools, and better treatment options [5, 6]. Succeeding in advancing these areas requires fresh approaches and ways of thinking, notably the development of better experimental models to study TB [7]. In this review, we discuss the different experimental approaches used in TB research, from in vivo models to human-based cell culture ones (Table 1). We also propose a road map of the available experimental approaches to study TB and of alternatives that are envisaged in a near future (Fig 1). We highlight the most recent advances in developing in vitro 3D models and introduce the potential of lung organoids as a new tool to study host—pathogen interactions during *Mycobacterium tuberculosis* infection. The development of such models requires a deep understanding of the disease pathogenesis and of the immune players, which are not the focus of this review and have been extensively reviewed elsewhere [8–10].

**Table 1:** Experimental models for the study of tuberculosis (TB).

Tools in TB research		Scientific potential	Limitations	Other considerations		
				Costs	Infrastructure requirements	Skills
**Animal models**	**Whole animal models** (nonhuman primates, rabbits, guinea pig, mouse, zebrafish)	Study of the immune response during Mtb infection in a whole organism, Genetic manipulation of key molecules and pathways, Better understanding of host—pathogen interactions	Anatomical differences, pathogenicity, and virulence of Mtb when compared to the human system, Difficulty to establish LTBI animal models, Some models limited by the lack of immunological-based tools, Limited housing capacity for larger animal models, Ethical, practical, and economic issues, Poor clinical outcome prediction	$ $ $ $	Appropriate animal housing, Animal Biological Safety Level 3 laboratories, Precise training	++++
**Human-based models**	**2D model** (cell lines)	Easily infected by Mtb with production of immune mediators, Lack of confounding factors, Study of Mtb cellular invasion and intracellular replication, Cell lines can be bought	Genetically transformed cells, Lack of tissue-like structure, Poor clinical outcome prediction	$ $	Biological Safety Level 3 laboratories, Precise training	++
	**2D model** (primary cells)		Require samples from patients, Lack of tissue-like structure, Poor clinical outcome prediction	$ $		++
	**3D model organoids** (pluripotent stem cells)	Primary tissue derived, Self-renewal and self-organization capacity, Lack of confounding factors, Tissue-like structure and function	Lack of the immune system, Absence of local microenvironment	$ $ $		+++

**Abbreviations:** Mtb, *Mycobacterium tuberculosis*; LTBI, latent tuberculosis infection

^$ $ $ $^, very high costs;

^$ $ $^, high costs;

^$ $^, intermediate costs;

^++++^, very high skills;

^+++^, high skills;

^++^, intermediate skills

## In vivo models in TB research

**Fig 2:**
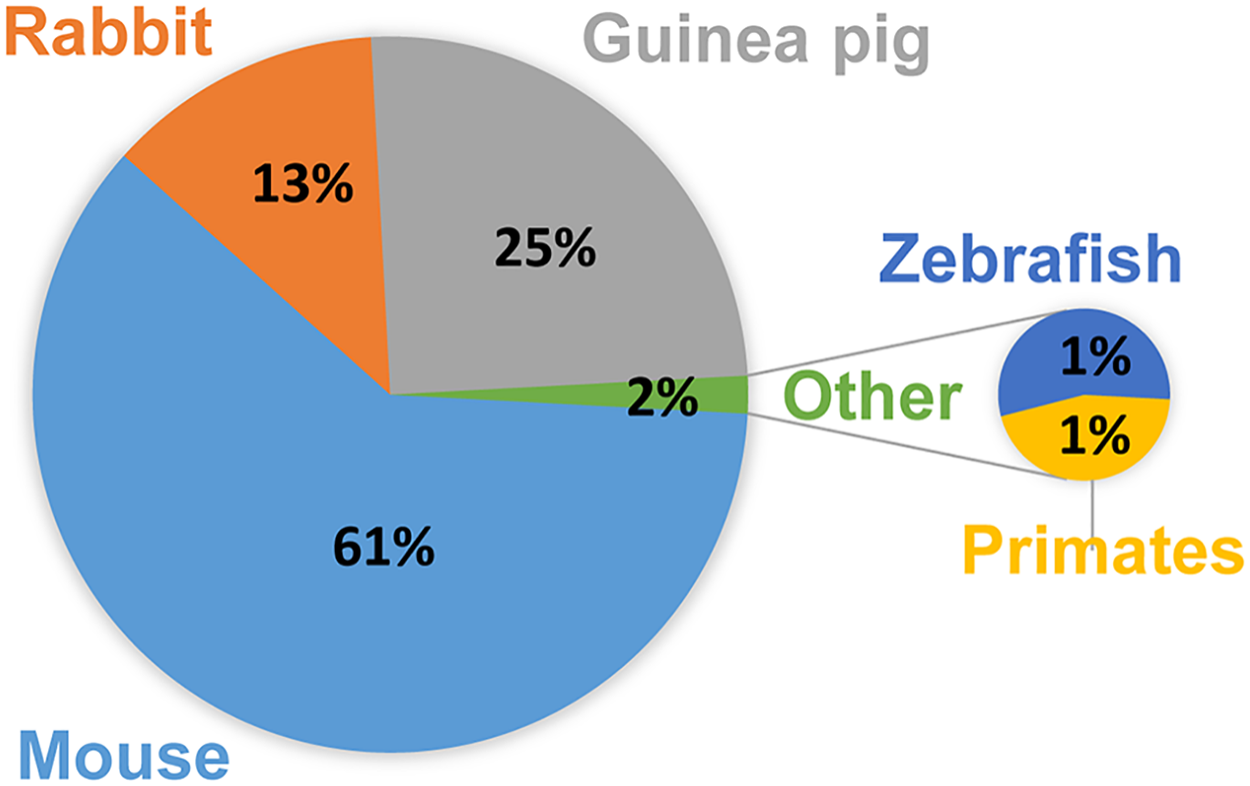
Proportion of different animal models in TB research. Pie chart illustrating the percentage of publications for each of the most commonly used animal models in TB research. Results from a Pubmed search performed on 9 February 2017 using the following key words: “mouse AND tuberculosis,” “guinea pig AND tuberculosis,” “rabbit AND tuberculosis,” “non-human primate AND tuberculosis,” and “zebra fish AND tuberculosis.” Percentages were calculated based on the total number of publications for all animal models.

Several animal models are used in TB research (Fig 2), ranging from zebrafish to nonhuman primates (NHPs) [11, 12]. Mice are preferred model animals for a number of practical reasons, such as availability of immunological-based tools for mice, the existence of genetically modified mouse strains, and the small size and cost-effectiveness of maintaining mice in the laboratory [13–15]. Whereas many important aspects of the immune system are indeed conserved, there are also important differences that hamper the use of the mouse model of infection in our understanding of TB pathogenesis. The mouse is not a natural host for *M*. *tuberculosis*, and lung cavitation, a key characteristic for the disease transmission in humans [16], is not observed for the 2 most-used mouse strains (Balb/c and BL6) [13]. Necrotizing responses to *M*. *tuberculosis* occur in other mouse strains [17], indicating the impact of genetic variability on the outcome of infection. A recent study illustrates this fact by demonstrating that the susceptibility to TB infection and the efficacy of Bacillus Calmette-Guerin (BCG) vaccination varied greatly when genetically different mouse strains were used [18]. It is thus not surprising that, depending on the mouse strain used, different studies report different data. Furthermore, variability in the reported results is enhanced by different experimental end points used [19, 20]. The route and dose of *M*. *tuberculosis* administration and the mouse microbiome are also thought to contribute to variable findings.

Since currently used mouse models fail to fully reflect human immunity to TB, several studies were performed using humanized mice. Humanized mice can be generated through the reconstitution of immunocompromised mice with human hematopoietic cells of different origins [21]. Infection of humanized mice with *M*. *tuberculosis* reproduced important hallmark features of human TB disease pathology, such as the formation of organized granulomatous lesions, caseous necrosis, and bronchial obstruction [22, 23]. However, abnormal T-cell responses and an impaired bacterial control were also observed [23]. In line with this, humanized mice generated by engraftment of human leukocyte antigen (HLA)-restricted cells showed partial function of innate and adaptive immune systems, culminating in antigen-specific T-cell responses to mycobacterial infection but also in lack of protection [24]. Other approaches consist in infecting transgenic mice expressing human-specific molecules such as, for example, the human cluster of differentiation group 1 CD1, which allows for the study of a humanized immune system using the mouse model of infection [25]. In all, humanized mice are a good tool to study TB, being particularly relevant for the study of HIV/TB, as recently shown [26]. However, this model requires further improvement to reach its full potential for TB research.

To address some of these limitations, other animal models have been used. For example, guinea pigs and rabbits may be considered better models to study the humanlike granuloma formation, a hallmark of *M*. *tuberculosis* infection in the lung [14, 27, 28], although they still fail to display other characteristics of the human disease. Additionally, they are much more difficult to maintain in the lab and a lot less immunology tools are available for these 2 species, which greatly limits their use. Infection of zebrafish (*Danio rerio*) embryos with the natural fish pathogen *M*. *marinum* is also used as a model for the study of granuloma formation [29–31]. Several similarities were found in the cellular and molecular events presiding *M*. *marinum* and *M*. *tuberculosis* infections [32–34], despite the many differences between these 2 diseases. Research on zebrafish embryos benefits from the similarities between *M*. *marinum* and *M*. *tuberculosis*, i.e., from the optical transparency of the embryos, which facilitates the use of advanced imaging techniques, and from the easy genetic manipulation of zebrafish, which allows for deep mechanistic molecular studies. Because zebrafish embryos lack a fully developed immune system, the study of later stages of infection requires the use of adult fish, thus abrogating the advantages of using embryos. Furthermore, the physiological differences between zebrafish and humans are enormous, which inevitably imposes some limitations to the use of this model. As for the other animal models, specific facilities for housing zebrafish are required. NHPs are so far considered as the best animal model for TB research [35, 36], as the disease pathogenesis parallels that observed in humans [37]. NHPs present lung cavitation [38]; show a spectrum of disease overlapping that of humans, namely, with the establishment of latent TB infection [38]; display a susceptibility to TB in the presence of comorbidities such as HIV and anti—tumor necrosis factor (TNF) treatment similar to that reported in humans [39, 40]; and present a transcriptomic signature of disease comparable to the human one [41]. However, the ethical, practical, and economic problems that are inherent to NHP research [36, 42], exacerbated when the animals are made to develop a potentially fatal infection, hinder the generalized use of this animal model, which in fact accounts for only 1% of the papers published in TB (Fig 2). In conclusion, important advances in our understanding of TB have been made through the use of different animal models. However, in addition to each model’s specific limitations, all animal-model research into human diseases is ultimately restricted by the need to translate findings across species. This calls for the wider use of human-based models to complement and reduce the use of experimental in vivo research.

## Human-based in vitro models in TB research

Owing to the central role of the macrophage as host and effector cell during *M*. *tuberculosis* infection [43, 44], many studies have been centered in macrophage cell cultures. In terms of human-based systems, monocyte-derived macrophages are the most widely used culture. Among these is the human monocytic leukemia cell line, THP-1, which is easy to culture, yielding a nearly unlimited amount of cells for experimental purposes. THP-1 cells are typically differentiated to macrophages through the stimulation with phorbol 12-myristate 13-acetate (PMA) for 3 days, although different protocols are found in the literature [45, 46], which may contribute to some variable findings. Macrophages can alternatively be freshly derived by extracting and culturing human peripheral blood mononuclear cells (PBMCs) in the presence of differentiating factors, namely, granulocyte-macrophage colony stimulating factor (GM-CSF) or macrophage colony stimulating factor (M-CSF) [47], or of human serum [48]. In these cases, the macrophages are of primary origin, but because of the in vitro differentiation process, their properties are most likely different from tissue-resident cells. Although alveolar macrophages would be ideally used, access to these cells is a costly procedure that requires lengthy ethical approvals, which limits their use. In vivo, *M*. *tuberculosis* is found in foamy macrophages. These cells result from pathogen-induced dysregulation of host lipid synthesis and sequestration and play a key role in both sustaining persistent bacteria and contributing to the tissue pathology [49]. Therefore, in vitro differentiation of foamy macrophages is an excellent tool for the study of macrophage-pathogen interactions. A protocol to convert cultured macrophages (THP-1 or primary) into foamy cells has been developed by incubating these cells under hypoxia [50]. Other alternatives for the differentiation of foamy cells include the exposure of cell cultures to palmitic acid, oleic acid, or lipoproteins [51] or to surfactant lipids [52].

Given the importance of working with primary, unmanipulated cells, many studies have been performed using freshly isolated human PBMCs [53]. Human PBMCs are easily accessible, cost-effective, and readily infected with *M*. *tuberculosis*, responding to the infection with the production of relevant immune mediators such as TNF and other interleukins as well as chemokines [53]. Furthermore, the PBMC response captures interactions between different immune cell types, such as monocytes, T cells, and B cells, which are in fact interacting during natural immune responses. However, these cells still differ from the tissue-resident ones and when used in in vitro cultures lack the environmental stimuli that ultimately shape cellular responses to infection. In addition to the standard monolayer cultures, PBMCs have been used to develop in vitro models of human mycobacterial granulomas. In 1 study [54], a sequential recruitment of human monocytes and lymphocytes towards mycobacterial antigen-coated artificial beads or live mycobacteria was observed. This recruitment culminated with the formation of a cellular structure reminiscent of natural mycobacterial granulomas in terms of morphology and cell differentiation [54]. This or similar/improved models have been used in several studies [55–57]. A different approach based on the culture of human PBMCs in a collagen matrix with a low dose of *M*. *tuberculosis* was used to develop an in vitro model of human TB granuloma with dormant bacteria [58]. This model recapitulated important characteristics of the mycobacterial granuloma, such as the aggregation of lymphocytes surrounding infected macrophages, the formation of multinucleated giant cells, the presence of secreted cytokines and chemokines in the culture supernatants, and the reactivation of *M*. *tuberculosis* upon immune suppression caused by TNF blockade [58]. These models offer the possibility of studying the infection by *M*. *tuberculosis* in a more physiological environment, resembling the structure of the infected human tissue. They constitute valuable approaches for the study of cell—cell interactions, cell differentiation, and bacterial control.

**Fig 3:**
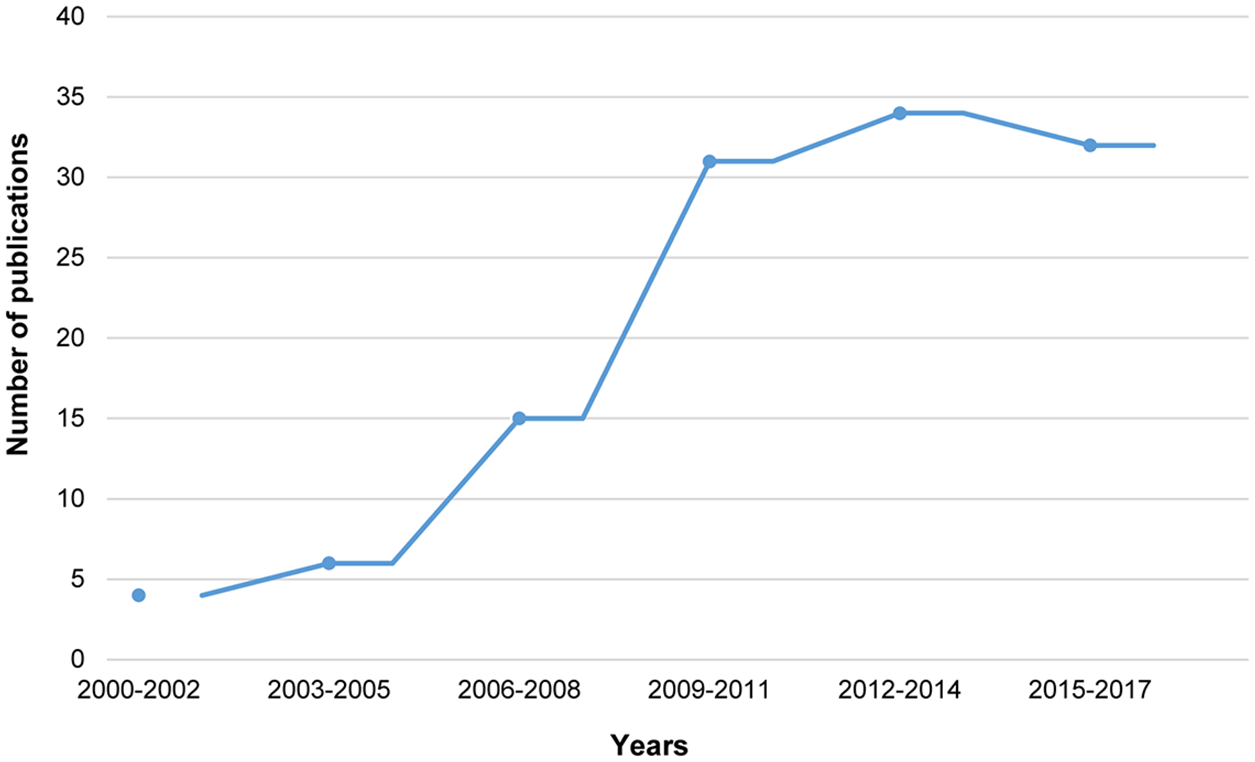
Use of 3D systems in TB research. Graph illustrating the increasing number of publications using 3D models for TB research between January 2000 and February 2017, every 3 years. Results are from a Pubmed search performed on 14 February 2017 using the keywords “3D models AND tuberculosis”.

To further reflect the complex environment and structure of the human lung, a growing body of studies are resorting to the use of new technologies in the tissue-engineering field to advance human-based TB research models into the 3D era (Fig 3) [59]. Tissue bilayer systems consisting of epithelial and endothelial cell layers were initially developed to study the early events of alveolar infection [60, 61]. More recently, through the use of these systems, microfold (M) cells were shown to play a critical role in translocating *M*. *tuberculosis* to initiate lung infection [62]. A study combining lung-derived epithelial cells and fibroblasts with peripheral blood primary macrophages reported the establishment of a lung tissue model that upon infection led to the clustering of macrophages reminiscent of early TB granuloma formation [63]. Similarly, another report showed the implementation of an in vitro human 3D lung tissue model to study *M*. *tuberculosis* infection that allowed the analysis of human granuloma formation and resembled some features of TB [64].

A novel bioengineering approach utilized bioelectrospray technology to generate microspheres of *M*. *tuberculosis*–infected human PBMCs in a 3D extracellular matrix [59, 65]. This model takes advantage of the high throughput potential of the bioelectrospray system and allows the interrogation of host—pathogen interactions in 3D in the context of an extracellular matrix [59, 66]. When combined with a microfluidic system to enable pharmacokinetic modeling, this model also showed great potential to monitor the efficacy of new antibiotic regimens or anti–*M*. *tuberculosis* drugs [65]. Although these experimental systems facilitate the discovery of the interactions between mycobacteria and host cells in a more physiological environment, they still bear some limitations, namely, the lack of vasculature and absence of other immune cells (e.g., neutrophils) that play a role in the multifaceted response in TB infection. Also, not all the models include epithelial and stromal cells, which are known to play important roles during infection [67, 68]. Finally, the spatial organization of the lung is mostly lost, and so is the role of the anatomical constraints during infection. The advances made in the development of all these models will certainly contribute to moving the field forward into novel strategies that overcome current limitations. In this context, other 3D and tissue-chip models are being explored.

## Organoids as infection models

Organoids are in vitro 3D cell cultures generated from embryonic stem cells (ESCs), induced pluripotent stem cells (iPSCs), or adult stem cells (aSCs) that functionally and structurally mimic the organ they model [69, 70]. This technology is emerging as a promising tool to study organ development and disease “in a dish” [69, 70]. The potential of organoids to study infectious processes has been increasingly demonstrated in many original papers and recently reviewed by Mills and Estes [71], with most examples coming from human gastric [72], brain [73, 74], and gut [75, 76] organoids. So far, lung organoids have not been explored as a model to study infection.

## Lung organoids in TB research

Human lung organoids have been generated through different technologies [77]. The most advanced studies involve the differentiation of human embryonic stem cells into endoderm cells, anterior foregut endoderm cells, lung progenitor cells, and, finally, various types of airway epithelial cells. If this procedure is performed in a 3D structure, a human lung organoid is formed, as initially described by Dye et al. [78] and Konishi et al. [79]. These relatively immature organoids may be transplanted into mice to complete their differentiation in an in vivo environment, into adultlike airways [80].

Despite some limitations, lung organoids recapitulate important features of the lung, such as heterogeneous cell composition, spatial organization, and retention of a stem cell population harboring the capacity for both self-renewal and differentiation [70]. There is increasing evidence that human lung organoids may be used to investigate the cellular and molecular pathways implicated in lung development and lung diseases as well as screening platforms for drugs directed at respiratory diseases [77]. At the disease level, the application of lung organoids to cancer development, cystic fibrosis, and infection is envisaged although still is unexplored in TB research. The obvious advantage of lung organoids over 2D and 3D cultures relies on their spatial organization and heterogeneity of the cellular components. As compared to the animal model, infection of lung organoids allows the inclusion of very early time points, which are difficult to follow in in vivo infections, whilst at the same time overcoming species differences and reducing the use of animals in research. Thus, as the lung organoid technology stands, human-derived lung organoids could be explored to study the early events of infection, namely, the initial interactions of *M*. *tuberculosis* with the lung epithelium [67]. Of the aforementioned experimental models, both 2D and 3D cultures based on PBMCs may also be explored to investigate the early immune events during infection, although to a lesser complexity than organoids.

Although there are indeed exciting perspectives for the use of lung organoids as a model for TB research, some important challenges remain before they can be more systematically used as experimental models. Chief among these is the introduction of immune cells in the structure of lung organoids. Only then will lung organoids cover the complexity of immune response and of the stromal-immune cells’ cross talk upon in vitro infection. Also, the introduction of the vasculature would be an important improvement to create a more dynamic model in which the microenvironment of an airway could be experimentally controlled. This dynamic lung organoid would be an interesting model for drug screening. In this context, microfluidic cell culture devices called “organs on a chip” have also generated airway epithelium from human adult airway cells grown on an air—liquid interface platform [81, 82]. Another important step forward would be the development of lung organoids from iPSCs instead of ESCs, as this will offer the possibility of including in the disease modelling individual variability, either genetic or caused by extrinsic conditions. In the context of TB research, this would allow for the study of host–*M*. *tuberculosis*–microenvironment interactions at an individual level by infecting lung organoids generated from individuals with HIV or diabetes versus controls or from smokers versus nonsmokers. This would be of utmost importance as the molecular mechanisms underlying the impact of comorbidities and life habits on the course of infection remain incompletely understood. Additionally, comorbidities are very difficult to incorporate in the other complex experimental system—the animal. Generation of personalized lung organoids would also open new avenues for the study of individual responses to therapies and thus for the implementation of personalized medicine.

## Conclusions

TB remains a devastating disease to mankind and a huge challenge for the scientific community. From many epidemiological studies, it is clear that the progression of the disease is highly related to the host immune status, and as such, a deep understanding of the immune response to *M*. *tuberculosis* is critical for the development of novel preventive and therapeutic strategies. However, the lack of experimental systems that parallel the complexity of the human disease remains a major gap hindering the in-depth study of the immune response in TB. Critical species differences mean that the widely used animal models only partly recapitulate the human disease. NHP models are the most representative ones but bear high operational and maintenance costs. Traditional human cellular systems overcome the interspecies translation problem but are limited by their low level of complexity and the abnormal characteristics of cell lines. Recent development of human-based tissue models is promising real alternatives for the experimental study of human TB. State-of-the art in vitro models have now incorporated several important characteristics of “real-life” tissues, namely, the presence of different cell types and of the extracellular matrix. Technological advances coupled to these models allow for the experimental manipulation of different variables, which is critical in studies of host—pathogen interactions or in drug-screening processes. A key next step will be to introduce in these models the anatomical constraint associated with the lung tissue. Albeit at very early days, lung organoids hold a great promise here. The road from lung organoids to complete lungs “in a dish” is still a long one, but creating a lung structure composed of different stromal cells and coupled with a competent immune system would unquestionably provide a major leap forward in TB research. Being able to use as starting points cells from different individuals (TB patients or latently infected people with different genetic backgrounds and comorbidities) would constitute a revolutionary way of studying TB. This would open many new avenues to investigate long-standing questions and put us in a privileged position to effectively tackle TB.

In sum, recent advances in tissue engineering and future steps in this area will certainly play an important role in the development of new tools for the study of infectious diseases. Such tools hold the potential to replace some animal experiments and overall lead to a reduction of the number of animals used in TB research. Most importantly, these tools will allow for a series of key questions to be answered in a more precise way by including individual variability at the single-cell and tissue levels.
